# *Aspergillus nidulans gfdB*, Encoding the Hyperosmotic Stress Protein Glycerol-3-phosphate Dehydrogenase, Disrupts Osmoadaptation in *Aspergillus wentii*

**DOI:** 10.3390/jof10040291

**Published:** 2024-04-16

**Authors:** Veronika Bodnár, Károly Antal, Ronald P. de Vries, István Pócsi, Tamás Emri

**Affiliations:** 1 Department of Molecular Biotechnology and Microbiology, Institute of Biotechnology, Faculty of Science and Technology, University of Debrecen, H-4032 Debrecen, Hungary; bveronika001@gmail.com; 2 Doctoral School of Nutrition and Food Sciences, University of Debrecen, H-4032 Debrecen, Hungary; 3 Department of Zoology, Eszterházy Károly Catholic University, Eszterházy tér 1, H-3300 Eger, Hungary; antalk2@gmail.com; 4 Fungal Physiology, Westerdijk Fungal Biodiversity Institute & Fungal Molecular Physiology, Utrecht University, 3584 CS Utrecht, The Netherlands; r.devries@wi.knaw.nl; 5 HUN-REN–UD Fungal Stress Biology Research Group, H-4032 Debrecen, Hungary

**Keywords:** *Aspergillus nidulans*, *Aspergillus wentii*, glycerol-3-phosphate dehydrogenase, hyperosmotic stress, RNA sequencing

## Abstract

The genome of the osmophilic *Aspergillus wentii*, unlike that of the osmotolerant *Aspergillus nidulans*, contains only the *gfdA*, but not the *gfdB*, glycerol 3-phosphate dehydrogenase gene. Here, we studied transcriptomic changes of *A. nidulans* (reference strain and Δ*gfdB* gene deletion mutant) and *A. wentii* (reference strain and *An-gfdB* expressing mutant) elicited by high osmolarity. *A. nidulans* showed a canonic hyperosmotic stress response characterized by the upregulation of the trehalose and glycerol metabolism genes (including *gfdB*), as well as the genes of the high-osmolarity glycerol (HOG) map kinase pathway. The deletion of *gfdB* caused only negligible alterations in the transcriptome, suggesting that the glycerol metabolism was flexible enough to compensate for the missing GfdB activity in this species. *A. wentii* responded differently to increased osmolarity than did *A. nidulans*, e.g., the bulk upregulation of the glycerol and trehalose metabolism genes, along with the HOG pathway genes, was not detected. The expression of *An-gfdB* in *A. wentii* did not abolish osmophily, but it reduced growth and caused much bigger alterations in the transcriptome than did the missing *gfdB* gene in *A. nidulans*. Flexible glycerol metabolism and hence, two differently regulated *gfd* genes, may be more beneficial for osmotolerant (living under changing osmolarity) than for osmophilic (living under constantly high osmolarity) species.

## 1. Introduction

Osmolarity is an important ecological factor for fungi. Efficient adaptation to hyperosmotic stress allows them to occupy habitats with permanently high osmolarity or tolerate fast and substantial changes in this environmental parameter caused by either reduction in water content or an increase in the concentration of osmotically active compounds (osmolytes) [1,2,3,4]. Efficient adaptation to osmotic stress has practical consequences as well. Fungi can contaminate foods and feeds or deteriorate art treasures or architectural materials, even if they have low water activity [5,6,7]. Opportunistic fungal pathogens can successfully adapt to the high osmolarity of lung mucus plugs in patients with cystic fibrosis, which can cause severe infections [8,9]. On the other hand, due to their good osmotolerance, several fungi can work efficiently under the high sugar and salt concentrations or low water activity of several industrial fermentations [10,11,12]. Moreover, by varying the osmolarity of the medium, the morphology, as well as the primary or secondary metabolism of fungi, can be modified to improve microbial biotechnological processes [13,14]. Manipulating the osmotolerance of strains also represent a promising way to enhance product formation or growth of the strains in industrial fermentations or increase the viability of spores during their long-term storage [12,15,16].

The accumulation of osmolytes (including K^+^; Pro and other amino acids; and polyols such as erythritol, arabitol, mannitol, and particularly, glycerol) is a common response to the increased osmolarity of the environment [17,18,19,20]. Not surprisingly, enzymes involved in glycerol biosynthesis, like glycerol 3-phosphate dehydrogenase (Gfd, an enzyme that reduces dihydroxyacetone phosphate to glycerol 3-phosphate, which is then dephosphorylated into glycerol) or glycerol dehydrogenase (Gld, an enzyme that produces glycerol by reducing dihydroxyacetone formed during the hydrolysis of dihydroxyacetone phosphate) frequently contribute to the osmotic stress response [18,20,21]. The evaluation of *Aspergillus* Gfd sequence data revealed that the ancient *gfd* gene was duplicated before the diversification of aspergilli, and consequently, most of the *Aspergillus* species, including *Aspergillus flavus*, *Aspergillus fumigatus*, *Aspergillus nidulans*, *Aspergillus niger*, *Aspergillus oryzae*, *Aspergillus sydowii*, or *Aspergillus terreus* have two *gfd* genes (*gfdA* and *gfdB*) [22]. The two Gfds differ from each other, not only in their sequence, but also in their regulation and function. Genome-wide transcription data revealed that *A. nidulans gfdB* was upregulated by menadione sodium bisulfite (MSB), or H_2_O_2_ elicited oxidative and NaCl induced osmotic stress, while *gfdA* showed some upregulation only under MSB stress [23]. Deletion of *gfdB* increased oxidative (diamide, *terc*-butylhidroperoxide, H_2_O_2_) and more moderately cell wall integrity (Congo Red) stress sensitivity of *A. nidulans*, but did not influence substantially the osmotic stress tolerance [24]. In contrast, deletion of *gfdA* caused strong reduction in growth (on glucose, but not on glycerol), which was remediable with addition of 1 M NaCl, and also increased cell wall integrity (Calcofluor White) stress sensitivity of the strain [25]. In *Aspergillus fumigatus*, deletion of *gfdA* also reduced significantly the growth on glucose and osmotic stress elicited by either NaCl or sorbitol could restore this growth defect [26]. The deletion of *gfdB* did not reduce growth substantially, and the overexpression of *gfdB* in a Δ*gfdA* mutant did not restore the growth defect of the mutant [26]. 

Interestingly, the osmophilic *Asperillus glaucus* and *Aspergillus wentii* have only a *gfdA* orthologue, which led us to speculate that the loss of *gfdB* is somehow causally connected to the osmophilic nature of these species [22]. GfdB may contribute to the degradation of glycerol after osmotic stress decreased, or it may be important only in the quick response to osmotic stress. The importance of such functions can be reduced at constantly high osmolarity. Deletion of either *gfdA* or *gfdB* did not change *A. nidulans* into an osmophile [24,25], but the *A. nidulans* Δ*gfdA* gene deletion mutant showed osmotic stress remediable growth reduction [25]. The expression of *A. nidulans gfdB* (with its own promoter) in *A. glaucus* increased oxidative (MSB, tBOOH, H_2_O_2_) and cell wall integrity (Congo Red) stress tolerance of the fungus, demonstrating the importance of GfdB in the protection against these stresses, but it did not affect the osmophilic nature of *A. glaucus* [27]. In contrast, the *A. wentii* strains expressing *A. nidulans gfdB* (with its own promoter) showed only a minor and sporadic improvement in their oxidative stress tolerance [28]. Although these strains did not grow better than the wild type strain at normal osmolarity, the growth-promoting effect of 2 M sorbitol or 1 M NaCl was significantly smaller in the case of the mutants, suggesting that the presence of GfdB disturbed the osmoregulation of *A. wentii* [28]. 

Here, we applied a transcriptomic approach to more completely understand the physiological consequences of the presence of *A. nidulans* GfdB in *A. wentii*. Using RNA sequencing (RNAseq), we recorded transcriptome changes under hyperosmotic stress in the wild type and *gfdB* gene deletion mutant (Δ*gfdB*) *A. nidulans*, as well as in the wild type and *An*-*gfdB* expressing *A. wentii* cultures. We used 2 M sorbitol, 1 M NaCl, or the combination of them (2 M sorbitol + 1 M NaCl) to treat the cultures. Both stressors are osmotically active compounds, frequently used in laboratory practice to induce hyperosmotic stress; however, the responses induced by them differ due to Na^+^ toxicity [29,30]. Comparing the genome-wide transcriptional changes, we concluded that it was not the loss of *gfdB* that initiated the development of osmophilic properties, but rather the development of osmophilic character which made the loss of this gene beneficial.

## 2. Materials and Methods

### 2.1. Strains and Culture Conditions 

The *A. nidulans* and *A. wentii* strains listed in Table 1 were used in this study. All strains were maintained on Barratt’s minimal agar plates [31] at 25 °C. Conidia, freshly harvested at 6 d, were used to inoculate the submerged cultures. 

Barratt’s minimal broth (100 mL in a 500 mL Erlenmeyer flask), inoculated with 1 × 10^8^ conidia, was incubated in a rotary shaker at 25 °C, at 220 rpm (approx. 3.7 Hz). *A. nidulans* mycelia were harvested after 36 h (THS30) or 38 h (Δ*gfdB*); meanwhile *A. wentii* mycelia were harvested after 65 h (both CBS141173 and ′c *gfdB*) of cultivation. Different incubation times were used in order to collect exponentially growing phases of mycelia with similar physiological conditions, in the case of each strain [28]. The collected mycelia were washed and then transferred into 100 mL fresh Barratt’s minimal broth or Barratt’s minimal broth supplemented with 2 M sorbitol, 1 M NaCl, and 1 M NaCl + 2 M sorbitol. The cultures were further incubated at 25 °C and 220 rpm, and samples were taken after 0.5 h (for RNA isolation) or 10 h (for characterizing the growth).

### 2.2. Reverse-Transcription Quantitative Real-Time Polymerase Chain Reaction (RT-qPCR) Assays

Lyophilized mycelia were used to isolate total RNA, according to the methods of Chomczynski [33] (“TRI reagent” method). RT-qPCR assays were carried out using the primer pairs listed in Table S1, employing an Xceed SG 1-step 2× Mix Lo-ROX qPCR Kit (Institute of Applied Biotechnologies, Prague, Czech Republic), following the manufacturer’s protocol. Relative transcription levels were characterized with the ∆CP (difference between the crossing point of the reference and the target gene within a sample) values using AN6542 (*actA*; *γ-actin*) for the *A. nidulans* samples and Aspwe1_0167845 (putative translation elongation factor EF-3) for the *A. wentii* samples as reference genes.

### 2.3. High Throughput RNA Sequencing

Total RNA was isolated from the lyophilized mycelia [33] from four different cultures (untreated, 2 M sorbitol, 1 M NaCl, and 2 M sorbitol + 1 M NaCl cultures) in the case of each strain (*A. nidulans* THS30 and ∆*gfdB*, *A. wentii* CBS141173, and ′c *gfdB*) using three biological replicates (48 samples in total). RNA sequencing (from library preparation to the generation of fastq.gz files) was carried out at the Genomic Medicine and Bioinformatic Core Facility, Department of Biochemistry and Molecular Biology, Faculty of Medicine, University of Debrecen, Debrecen, Hungary. A TruSeq RNA Sample preparation kit (Illumina, Praha, Czech Republic) was used for library preparation, according to the manufacturer’s protocol. A single-read 75 bp Illumina sequencing was performed in one lane of a sequencing flow cell on an Illumina HiScan SQ instrument (Illumina, San Diego, CA, USA), and 12–60 million reads per sample were obtained. The hisat2 (version 2.1.0; [34]) software was used for mapping the raw reads to the reference genomes (84–96% of reads were successfully aligned) and generating bam files, while featureCounts (Version 2.0.0, [35]) was used for calculating the read counts. The following genome and gff files were used (FungiDB; https://fungidb.org/fungidb/app, accessed on 25 August 2023): https://fungidb.org/common/downloads/release-65/AnidulansFGSCA4/fasta/data/FungiDB-65_AnidulansFGSCA4_Genome.fasta, accessed on 25 August 2023; https://fungidb.org/common/downloads/release-65/AnidulansFGSCA4/gff/data/FungiDB-65_AnidulansFGSCA4.gff, accessed on 25 August 2023; https://fungidb.org/common/downloads/release-65/AwentiiDTO134E9/fasta/data/FungiDB-65_AwentiiDTO134E9_Genome.fasta, accessed on 25 August 2023; https://fungidb.org/common/downloads/release-65/AwentiiDTO134E9/gff/data/FungiDB-65_AwentiiDTO134E9.gff, accessed on 25 August 2023. 

Differential expression analysis of the read counts was performed using DESeq2 (version 1.36.0; [36]). The “rpkm” function of the edgeR package (version 3.18) [37] was used to calculate the RPKM (reads per kilobase per million mapped reads) values, and the “prcomp” function was applied to perform PCA (principal component analysis). In the case of the *gfdB* gene (which is not part of the *A. wentii* CBS141173 genome), we counted the reads matching the sequence of the *A. nidulans gfdB* gene, available at FungiDB (https://fungidb.org/fungidb/app, accessed on 5 October 2023), using the BBmap software (version 39.01; https://sourceforge.net/projects/bbmap/, accessed on 5 October 2023) using “perfectmode” settings and utilized these counts to calculate all *gfdB* RPKM values. Note that in the case of the *A. nidulans* THS30 strain, the difference between the RPKM values calculated with data generated by the BBmap software and the values calculated using the “rpkm” function of the edgeR package (version 3.18) (data are available at GSE255841; Gene Expression Omnibus; http://www.ncbi.nlm.nih.gov/geo/, accessed on 31 January 2024) was less than 10%.

### 2.4. Evaluation of the Transcriptome Data

Upregulated and downregulated genes were defined as differentially expressed genes (DEGs; adjusted *p*-value < 0.05) where log_2_FC > threshold, or log_2_FC < -threshold, respectively, using threshold 1. FC stands for fold change value, and log_2_FC values were calculated by the DESeq2 software (version 1.36.0) using the untreated cultures (when the effect of osmotic stress treatment on a strain was studied), the cultures of the *A. nidulans* THS30, or the *A. wentii* CBS141173 strains (when the consequences of the genetic manipulation under one culturing condition was studied in the appropriate species) as references. 

Gene set enrichment analyses were performed on the ShinyGO V0.77 (http://bioinformatics.sdstate.edu/go77/, accessed on 5 September 2023) platform (*A. nidulans*) or on the FungiDB (release 66; https://fungidb.org/fungidb/app, accessed on 5 September 2023) platform (*A. wentii*), applying default settings. GO and KEGG pathway terms containing less than three genes, or hits with only one gene, were omitted from the analysis, and only hits with a corrected *p*-value (Benjamini–Hochberg correction) < 0.05 were taken into consideration during evaluation. Since gene set enrichment analyses are highly dependent on the size of the studied gene sets, the analyses for differentially expressed genes (DEGs) were performed with thresholds of 0 and 2.

Similarities in the changes of transcriptomes were characterized by either the Pearson’s correlation coefficient between the log_2_FC values of all genes of the compared two genome-wide transcriptional changes or the Venn-analyses of the upregulated and downregulated genes.

Genes regarded to be particularly important under osmotic stress (and upregulated by osmotic stress) in *A. nidulans* were collected from the papers cited in Refs. [38,39,40,41,42,43] and were regarded as “osmotic stress genes” here for clarity, even though they contribute to other stress responses as well. Genes encoding or putatively encoding enzymes with observed/predicted catalase, cytochrome c peroxidase, glutathione disulfide oxidoreductase, glutathione peroxidase, glutathione transferase, (heme) peroxidase, peroxiredoxin, superoxide dismutase, and thioredoxin-disulfide reductase activities according to FungiDB (https://fungidb.org/fungidb/app, accessed on 5 September 2023) were also collected and regarded here as “oxidative stress” genes. The “glycerol metabolism” gene set was created according to the method of Fillinger et al. [25]. Orthologues of these genes in *A. wentii* were collected from FungDB (https://fungidb.org/fungidb/app, accessed on 5 September 2023). When more than one *A. wentii* orthologue was listed in the FungiDB, the “best hit” among them was determined using the Blastp platform (https://blast.ncbi.nlm.nih.gov/Blast.cgi?PAGE=Proteins, accessed on 5 September 2023). The Fisher’s exact test (“fisher.test” function of R project; https://www.r-project.org/, accessed on 25 August 2023) was used to study the enrichment of these genes (*A. nidulans* genes, *A. wentii* orthologues, only “best hits” of *A. wentii* orthologues) in the appropriate gene sets.

## 3. Results

### 3.1. Deletion of gfdB in A. nidulans Produced Negligible Effect on Osmotolerance, While Expression of A. nidulans gfdB in A. wentii Reduced Growth, Irrespectively of the Osmolarity

The effects of sorbitol (2 M), NaCl (1 M), and NaCl + sorbitol (1 M and 2 M, respectively) treatments on *A. nidulans* THS30 (*gfdB*^+^, reference strain), *A. nidulans* Δ*gfdB* (*gfdB*^−^ mutant), *A. wentii* CBS141173 (*gfdB*^−^, reference strain), and *A. wentii* ′c *gfdB* (*gfdB*^+^, *An-gfdB* expressing mutant) cultures were studied.

The genome-wide transcriptional changes induced by the osmolites in the cultures of the four strains detected with RNAseq showed good correlation with the RT-qPCR data (Table S2). The Pearson’s correlation coefficients were higher than 0.8 for each gene studied, and for the whole dataset, they were 0.797 (*A. nidulans*) and 0.876 (*A. wentii*) (Table S2). According to the principal component analyses (Figure S1), stress treatments have a substantial effect on the transcriptomes, and the three biological replicates showed similar transcriptomes in each treatment with each strain, with the exception of sorbitol treated *A. wentii* CBS141173 and NaCl treated *A. wentii* ′c *gfdB* cultures, for which the similarity was reduced. Note that the long cultivation time of the osmophilic *A. wentii* CBS141173 strain under normal osmolarity before the treatments necessarily enhanced the possibility of increased within-group variations in these experiments.

**Figure 1:**
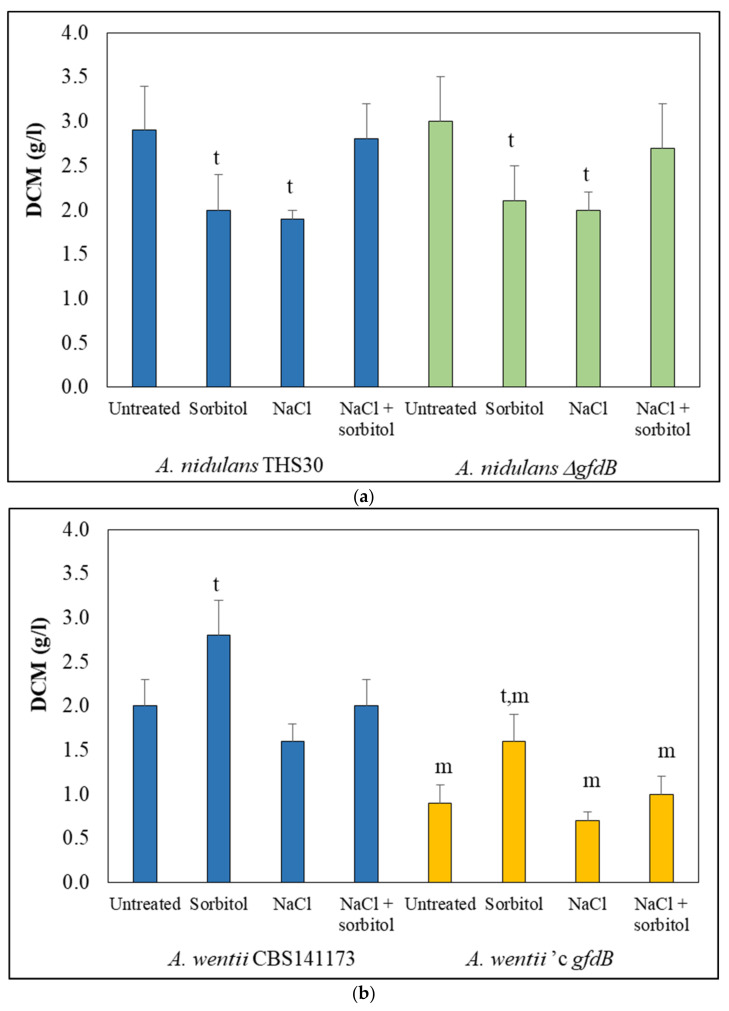
Effects of stress treatments on the growth of *A. nidulans* (**a**) and *A. wentii* (**b**) cultures. Growth was characterized by the dry cell mass (DCM) of the cultures. The mean ± SD of three biological replicates are presented. The letter “t” marks a significant effect (Student’s *t*-test, *p* < 0.05) of treatment (vs. untreated); “m” marks a significant effect of mutation (vs. same treatment in the reference strain).

**Figure 2:**
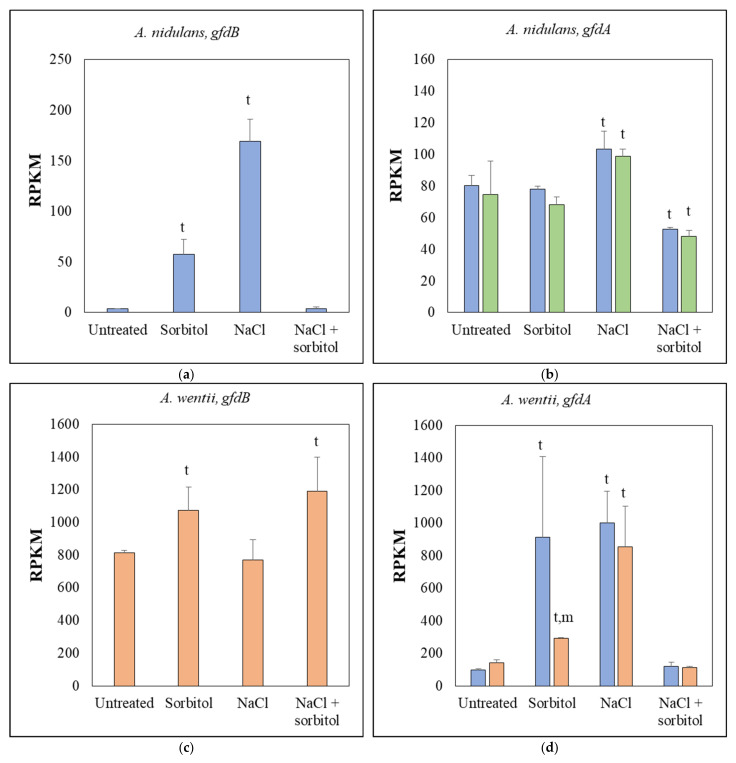
The effects of stress treatments on the transcription of the *gfdB* (**a**,**c**) and *gfdA* (**b**,**d**) genes in the wild type (blue) and mutant (Δ*gfdB*, green, or ′c *gfdB*, orange) strains of *A. nidulans* (**a**,**b**) and *A. wentii* (**c**,**d**). The mean ± SD of RPKM values from three biological replicates are presented. Significant (Student’s *t*-test, *p* < 0.05) differences between the stress treated and untreated cultures, or the mutant and the reference strains, are marked with the letters “t”, and “m”, respectively. For the *gfdA* gene, the “rpkm” function of the edgeR package, with default settings [37], was used, while for the *gfdB gene*, the BBmap software (version 39.01; https://sourceforge.net/projects/bbmap/, accessed on 5 October 2023), with “perfectmode” settings, was applied to calculate the RPKM values.

Sorbitol and NaCl stress treatments reduced the growth of *A. nidulans* THS30 in the submerged cultures (Figure 1a), as was expected from agar plate experiments [28]. In line with previous studies [28], *gfdB* gene deletion had no significant effect on growth (Figure 1a). Nor did the combined stress treatment (NaCl + sorbitol) significantly reduce the growth (Figure 1a). The *gfdB* gene was significantly upregulated by both sorbitol and NaCl stresses, in line with the results of previous studies [44], but this was not the case under the combined NaCl + sorbitol stress in the THS30 strain (Figure 2a). In contrast, the expression of *gfdA* changed less relative to *gfdB*, and these changes were specific for the type of osmotic stress. Transcription of the *gfdA* gene was slightly upregulated by NaCl treatment, slightly downregulated by the combined NaCl + sorbitol treatment, and remained unchanged in the presence of sorbitol in both strains (Figure 2b). These data suggest that in addition to GldB (NADP-dependent glycerol dehydrogenase), a key enzyme responsible for osmotic tolerance in *A. nidulans* [21] and GfdA [25], GfdB is also involved in the osmotic stress response. However, GfdB was less important ([24] and Figure 1a) than GldB, and its regulation and thus, its function, was different from that of GfdA.

When we applied the same stress treatments to *A. wentii* CBS 141173, its growth, in line with its osmophilic nature, either increased (on sorbitol) or did not change significantly (Figure 1b). The expression of the *An-gfdB* gene in the fungus did not substantially alter this pattern, but reduced growth significantly (Figure 1b). The *An-gfdB* gene (with its own promoter) was active in *A. wentii* ′c *gfdB* (Figure 2c) and showed an expression pattern different from that in *A. nidulans* (Figure 2a): it was slightly upregulated by sorbitol and NaCl + sorbitol treatment, but not by NaCl treatment. The *gfdA* gene behaved similarly to the *gfdB* gene in *A. nidulans* (Figure 2a) in both *A. wentii* strains (Figure 2d): the sorbitol and NaCl treatments, but not the NaCl + sorbitol treatment, caused upregulation, suggesting that *A. wentii* GfdA may be a functional orthologue of *A. nidulans* GfdB in osmoregulation. Interestingly, the expression of *gfdA* in *A. wentii* ′c *gfdB* lagged behind that of the CBS141173 strain when treated with sorbitol (Figure 2d). 

### 3.2. RNAseq Data Revealed That the Absence of GfdB Caused Only Minor Changes in the Osmotic Stress Response of A. nidulans

**Figure 3:**
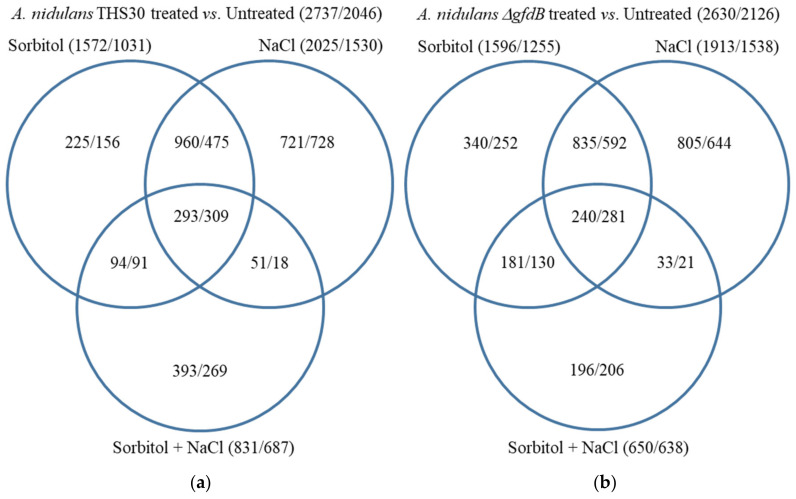

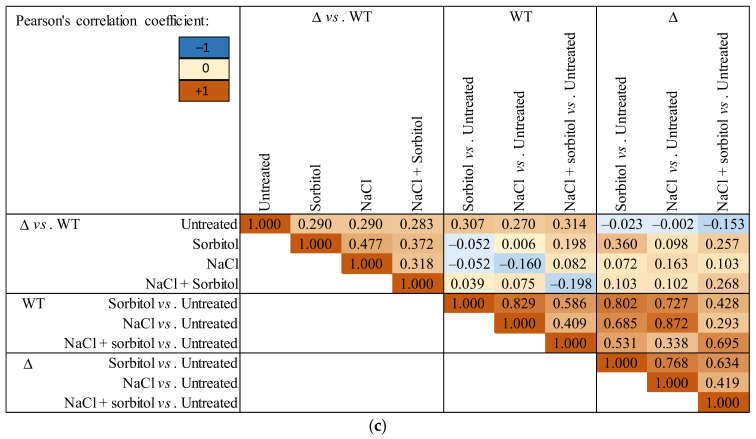
Genome-wide transcriptional changes in *A. nidulans* caused by stress treatments. Panels (**a**,**b**): Venn analyses of upregulated and downregulated genes (|log2FC| > 1) in the THS30 and the Δ*gfdB* strains. Panel (**c**): Pearson’s correlation coefficients between the log_2_FC values of the transcriptional changes. The THS30 and Δ*gfdB* strains are abbreviated as “WT” and “Δ”, respectively.

The upregulation of 2737 and the downregulation of 2046 genes were observed in at least one stress treatment in the *A. nidulans* THS30 strain (Figure 3a). The large overlap between the stress responsive genes of the sorbitol and NaCl treatments (Figure 3a), as well as the high correlation coefficient (Figure 3c) between the genome-wide transcriptional changes elicited by these two treatments, showed that the sorbitol and NaCl stress responses were similar. The response to the combined NaCl + sorbitol stress treatment was also similar to that of the two other stress responses (Figure 3a,c). However, this treatment caused only small transcriptional changes relative to the single stressor treatments (Figure 3a), which is consistent with the growth profile of the strain (Figure 1a) and the transcription profile of the *gfdB* gene (Figure 2a).

**Figure 4:**
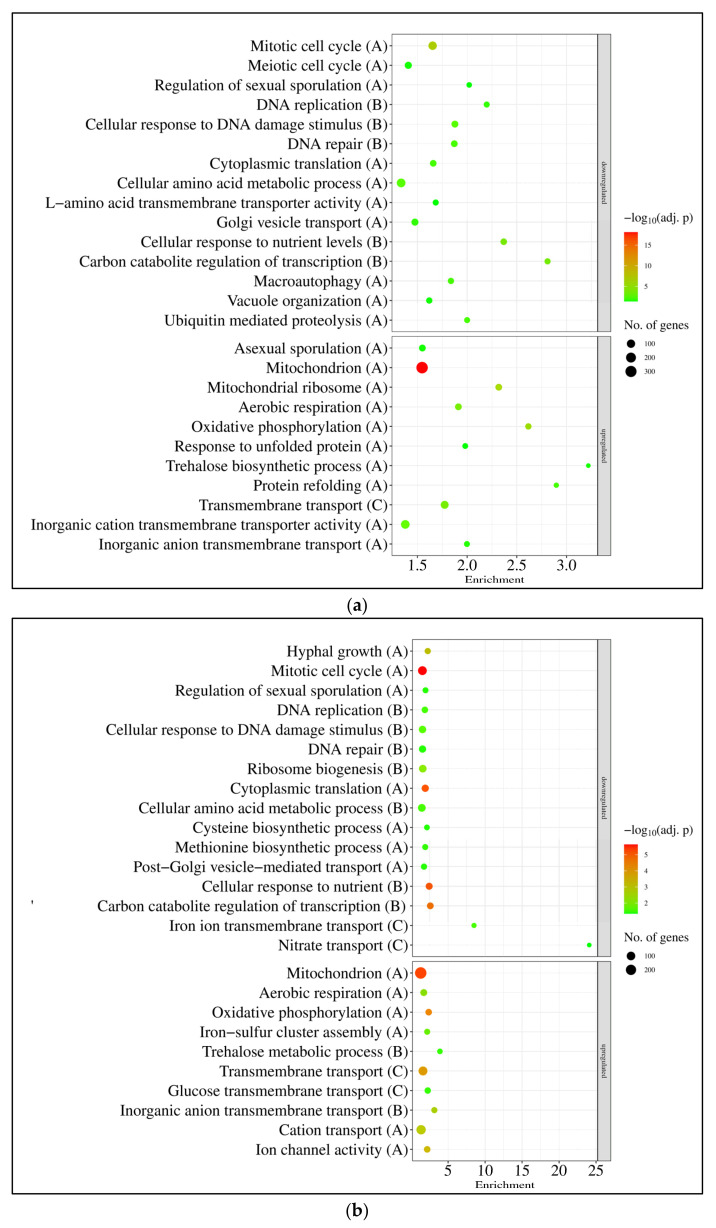

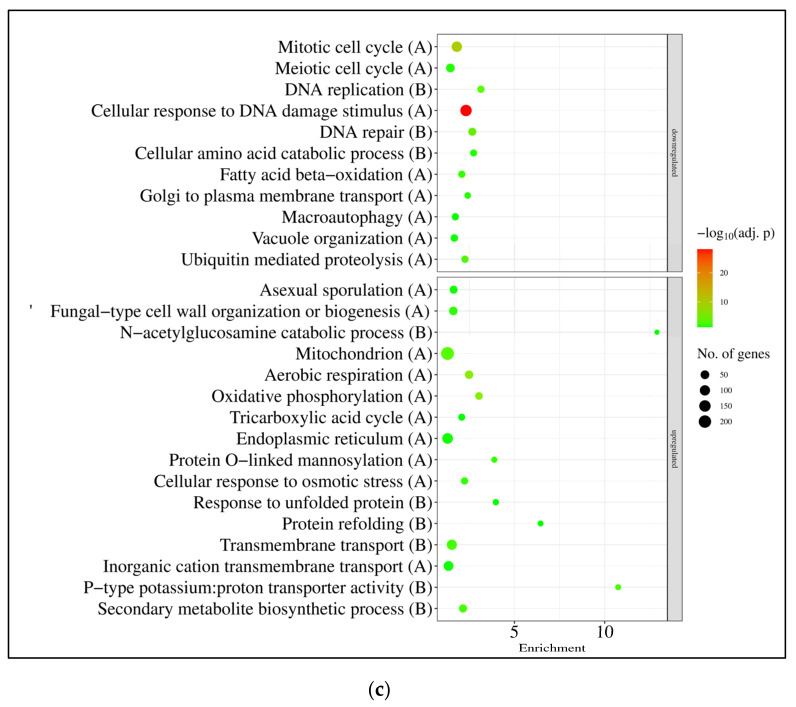
Gene set enrichment analyses of the effect of sorbitol (**a**), NaCl (**b**), and NaCl + sorbitol (**c**) treatments on *A. nidulans* THS30. Selected significantly enriched (*p* adjusted < 0.05) GO and KEGG pathway terms are presented. The full list of the enriched terms are available in Table S3. Letters in parentheses indicate the studied gene set: “A”—all DEGs; “B”—DEGs with |log_2_FC| > 1; “C”—DEGs with |log_2_FC| > 2. If a selected term was enriched in more than one gene set, only the set with the strongest criteria is presented.

Gene set enrichment analyses also demonstrated that there was a substantial overlap among the three responses (Table S3, Figure 4). The mitotic cell cycle, DNA replication and DNA repair genes, together with amino acid metabolism genes, were enriched in the downregulated gene set, while the mitochondrial function genes (including respiration and oxidative phosphorylation genes) and genes involved in various transport processes and ion homeostasis were enriched in the upregulated gene set in the case of each treatment (Table S3, Figure 4). Genes involved in sexual development (or meiotic cell cycle) also showed downregulation in each treatment, which was accompanied by the upregulation of asexual reproduction genes in the sorbitol and NaCl + sorbitol treatments (Table S3, Figure 4). In the case of single stressor (sorbitol, and NaCl) treatments, genes involved in bulk protein translation and carbon/glucose repression were also enriched in the downregulated gene sets, while the upregulated gene sets were enriched with trehalose metabolism genes (Table S3, Figure 4). In the presence of sorbitol (sorbitol and NaCl + sorbitol treatments), autophagy and protein degradation genes, as well as protein refolding genes, were enriched in the downregulated and upregulated gene sets, respectively (Table S3, Figure 4). Based on the literature, 16 genes were selected as those commonly upregulated under osmotic stress (Table S4; “osmotic stress genes”), [38,39,40,41,42,43]. The majority of these showed upregulation in our experiments under sorbitol and NaCl stresses (Table S4). A few of these genes were also upregulated under the combined stress treatment; however, their enrichment was not significant in the upregulated gene sets (Table S4). The transcriptional activity of most “glycerol metabolism” genes increased significantly when treated with sorbitol or NaCl, including genes for glycerol transport, biosynthesis, and degradation (Table S4). Since osmotic stress may also cause oxidative stress, and the deletion of *gfdB* increased oxidative stress sensitivity [24], the transcriptional behavior of antioxidant enzyme genes was also investigated. Sorbitol and NaCl treatments significantly increased the transcription of several antioxidative enzyme genes, including *sodB* (encoding mitochondrial Mn-superoxide dismutase [45], *catA* (encoding conidium specific catalase [46]) and *ccp1* (encoding cytochrome c peroxidase [47]); however, enrichment of this gene set among the upregulated genes was significant only under NaCl stress (Table S4).

**Figure 5:**
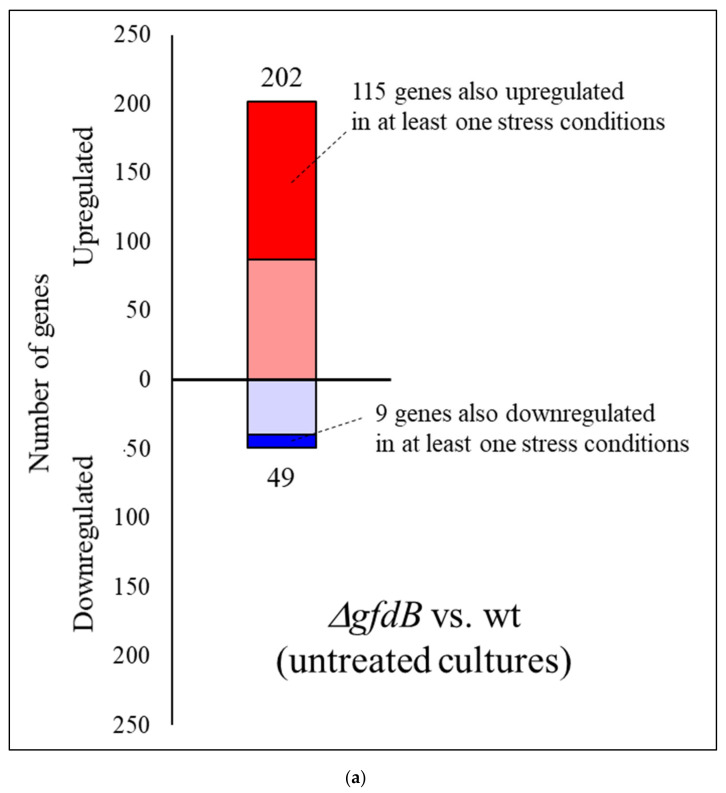

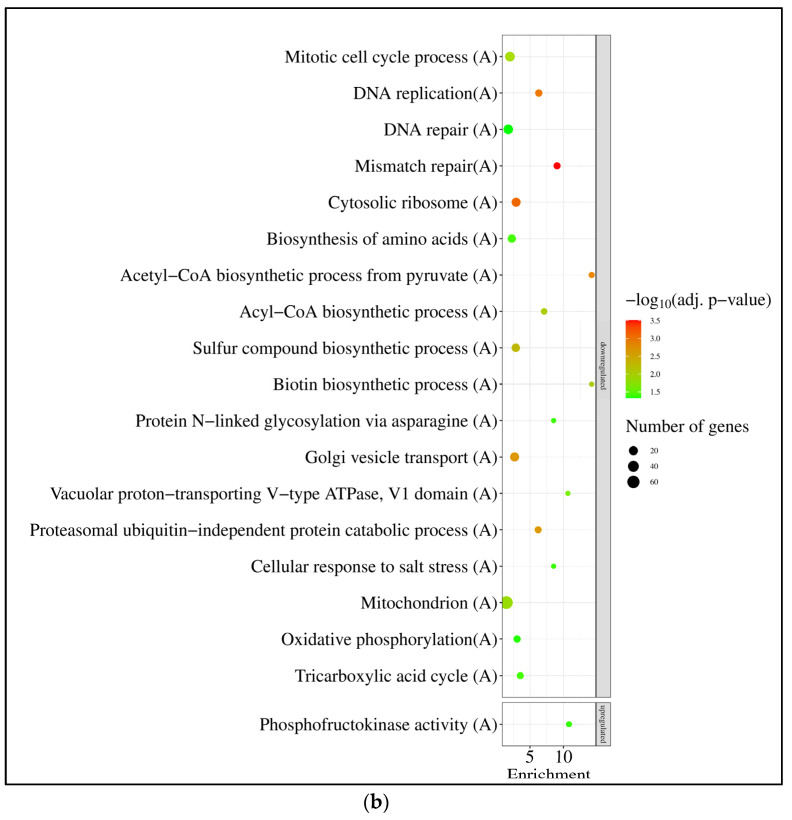
Direct comparison of the transcriptomes of *A. nidulans* THS30 and Δ*gfdB* strains under untreated conditions. The number of upregulated (red) and downregulated (blue) genes (DEGs with |log_2_FC| > 1) are presented from the comparison of the Δ*gfdB* mutant vs. the THS30 strain in untreated cultures (**a**). Gene set enrichment analyses of the consequences of gfdB gene deletion under untreated conditions (**b**). Selected significantly enriched (*p* adjusted < 0.05) GO and KEGG pathway terms are presented. The full list of the enriched terms are available in Table S3. Letters in parentheses indicate the studied gene set: “A”—all DEGs; “B”—DEGs with |log_2_FC| > 1; “C”—DEGs with |log_2_FC| > 2. If a selected term was enriched in more than one gene set, only the set with the strongest criteria is presented.

Deletion of the *gfdB* gene did not substantially alter the number of stress responsive genes: 2630 showed upregulation, and 2126 showed downregulation in at least one treatment (Figure 3b). Their distribution among the three treatments and the correlations between the three stress responses showed similar tendencies to those observed in the reference strain (Figure 3). The direct comparison of the transcriptomes of the Δ*gfdB* mutant and the reference strain revealed only small differences under untreated conditions (Figure 5a). Moreover, the overlap between the stress responsive gene sets of the two strains was also high (more than or close to 50% of the stress responsive genes) in each treatment (Figure S2). Accordingly, the stress responses of the Δ*gfdB* gene deletion mutant were similar to those of the reference strain. The results of gene set enrichment analyses also show that similar biological functions were regulated in the three stress responses of the mutant when compared with the results for the reference strain (Figure S3, Tables S3 and S4).

Deletion of the *gfdB* gene resulted in the upregulation of 202 genes and the downregulation of 49 genes under untreated conditions in the gene deletion mutant relative to the reference strain (Figure 5a, Table S5).

Genes that showed upregulation in at least one stress treatment in the THS30 reference strain were significantly enriched in the upregulated gene set (115 genes out of the 202 genes) (Figure 5a, Table S5). Genome-wide transcriptional differences found between the *gfdB* gene deletion mutant and the reference strain also showed some positive correlation with the stress treatment-elicited transcriptional changes in the reference strain (Figure 3c). Gene set enrichment analyses revealed some similarities between the transcriptional consequences of *gfdB* gene deletion and the stress treatments: as was the case for the stress treatments, the deletion of the *gfdB* gene downregulated the mitotic cell cycle, replication, and translation, as well as the repair and protein degradation genes (Figure 5b, Table S3). However, unlike the results for the stress treatments, it also downregulated genes related to mitochondrial processes like oxidative phosphorylation and tricarboxylic acid cycle genes (Figure 5b, Table S3), and substantial changes in the transcription of the osmotic stress, glycerol metabolism, and oxidative stress genes were not observed (Table S4). 

### 3.3. Expression of A. nidulans gfdB Substantially Disturbed the Physiology of A. wentii

**Figure 6:**
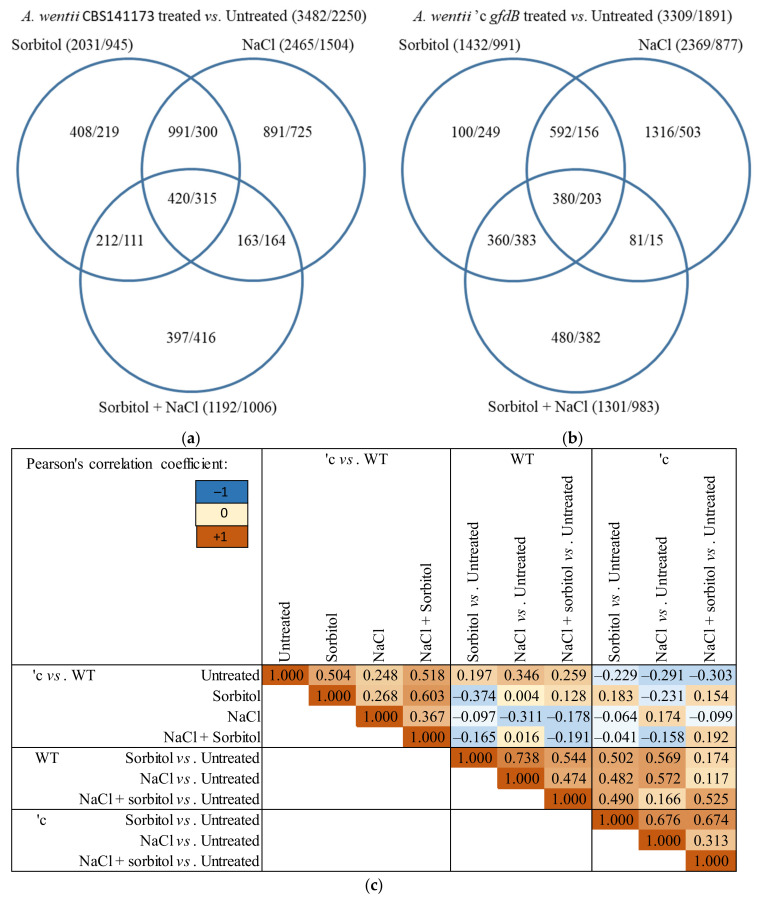
Genome-wide transcriptional changes caused by stress treatments in *A. wentii.* Panels (**a**,**b**): Venn analyses of upregulated and downregulated genes (|log2FC| > 1) in the CBS141173 and the ′c *gfdB* strains. Panel (**c**): Pearson’s correlation coefficients between the log_2_FC values of the transcriptional changes. The CBS141173 and ′c *gfdB* strains are abbreviated as “WT” and “′c”, respectively.

The applied stress treatments resulted in the upregulation of 3482 and the downregulation of 2250 genes in the *A. wentii* CBS141173 reference strain (Figure 6a). The large overlap between the stress responsive gene sets of the sorbitol and NaCl treatments (Figure 6a), as well as the high correlation coefficient (Figure 6c) between the genome-wide transcriptional changes elicited by these two treatments showed that the sorbitol and NaCl stress responses were similar. Again, the combined stress caused smaller transcriptional changes relative to the single stressor treatments (Figure 6a). 

**Figure 7:**
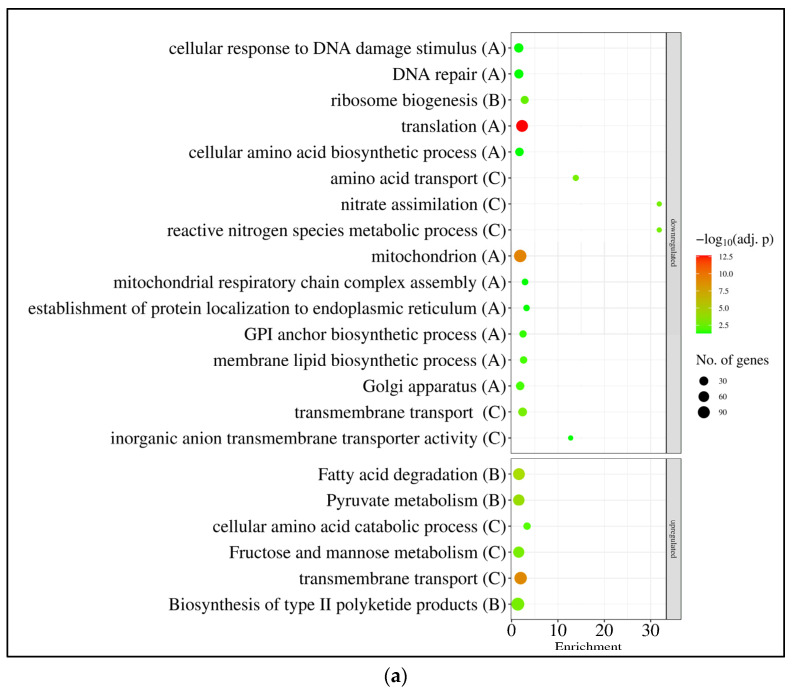

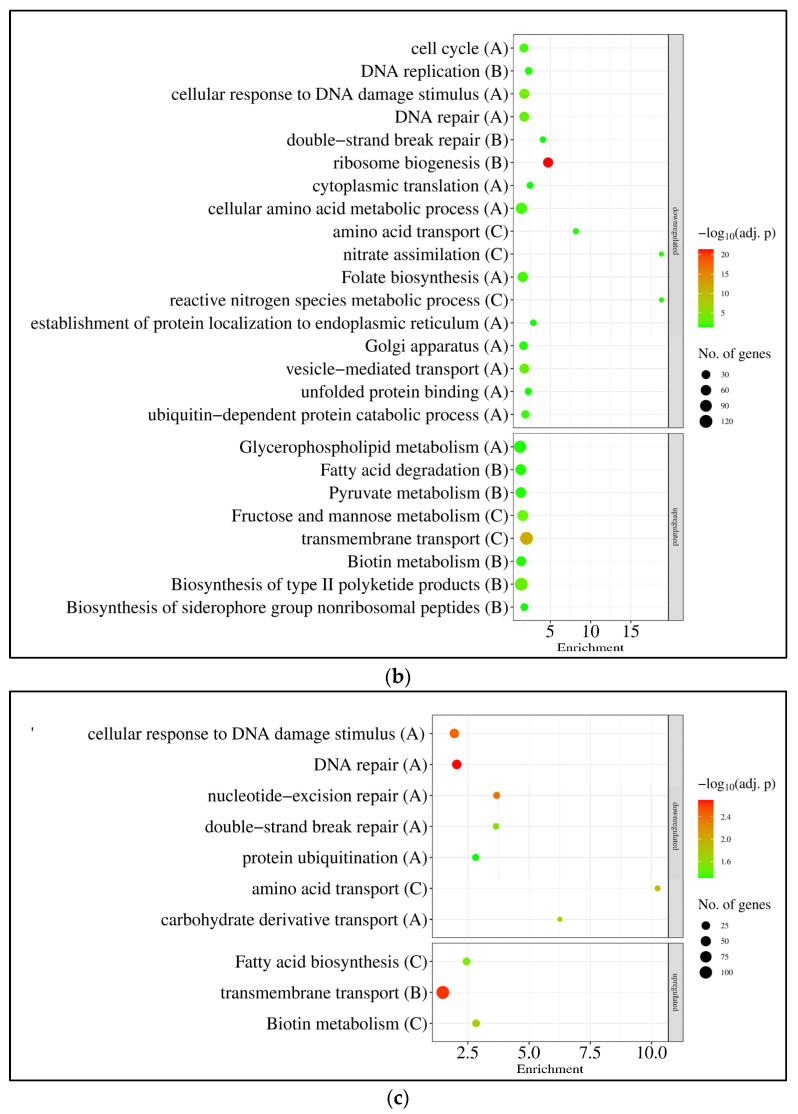
Gene set enrichment analyses of the effect of sorbitol (**a**), NaCl (**b**), and NaCl + sorbitol (**c**) treatments on *A. wentii* CBS141173. Selected significantly enriched (*p* adjusted < 0.05) GO and KEGG pathway terms are presented. The full list of the enriched terms are available in Table S3. Letters in parentheses indicate the studied gene set: “A”—all DEGs; “B”—DEGs with |log_2_FC| > 1; “C”—DEGs with |log_2_FC| > 2. If a selected term was enriched in more than one gene set, only the set with the strongest criteria is presented.

According to the gene set enrichment analyses, DNA repair and amino acid metabolism genes were enriched in the downregulated genes, while transporter genes were enriched in the upregulated gene sets, irrespectively of the applied treatments (Figure 7, Table S3). The ribosome biogenesis and translation genes were enriched in the downregulated gene sets, while the amino acid and fatty acid degradation genes, the fructose–mannose metabolism genes, and genes involved in secondary metabolism were enriched in the upregulated gene sets after sorbitol and NaCl treatments (Figure 7, Table S3). 

In line with the growth profile of the cultures (Figure 1b), DNA replication and the cell cycle genes showed bulk downregulation only in the NaCl treated cultures (Figure 7, Table S3). The mitochondrion function genes, which were upregulated by all osmotic stress treatments in *A. nidulans* THS30 (Figure 4, Table S3),were enriched in the downregulated gene set after the growth-promoting sorbitol treatment in *A. wentii* CBS141173 (Figure 4, Table S3). The orthologues of *A. nidulans* “osmotic stress genes” did not indicate an obvious upregulation in *A. wentii* as was shown in *A. nidulans* (Table S4). However, some genes showed strong upregulation after both sorbitol and NaCl treatments: besides *gfdA* (Table S4, Figure 2d), these included the *atfA* b-ZIP transcription factor gene orthologue ASPWEDRAFT_37015 and the *ypdA* phosphotransfer regulatory protein gene orthologue ASPWEDRAFT_99233 (Table S4). As in *A. nidulans*, some of the antioxidative enzyme genes were upregulated in the sorbitol or NaCl treated cultures of *A. wentii* CBS141173 (Table S4). Among the upregulated genes, ASPWEDRAFT_506345 (putatively encoding a superoxide dismutase), ASPWEDRAFT_48635 and ASPWEDRAFT_53830 (putatively encoding catalases), as well as ASPWEDRAFT_235979 (putatively encoding a cytochrome c peroxidase) are notable (Table S4).

Expression of the *An-gfdB* gene in *A. wentii* did not substantially alter the number of stress responsive genes: 3309 showed upregulation, and 1891 showed downregulation (Figure 6b). Their distribution among the treatments showed a more or less similar pattern to that found in the reference strain (Figure 6b). The overlap between the stress responsive gene sets in the three treatments and the pairwise correlation between the three transcriptional changes showed some decrease in the mutant relative to the reference strain, but they were still substantial (Figure 6). In view of the results of the gene set enrichment analyses, the three stress responses of the *A. wentii* ′c *gfdB* mutant strain were also more or less similar: cell cycle, DNA replication, DNA repair, ribosome biogenesis, protein degradation genes were enriched in the downregulated gene sets, while biotin and fatty acid biosynthesis, as well as transporter genes, were enriched in the upregulated gene sets (Figure S5 and Table S3). The fructose–mannose metabolism genes and secondary metabolism genes were enriched in the upregulated gene sets of the sorbitol and NaCl treated cultures, while the mitochondrial function genes were only enriched in the upregulated gene set of the NaCl + sorbitol treated cultures (Figure S5 and Table S3). 

**Figure 8:**
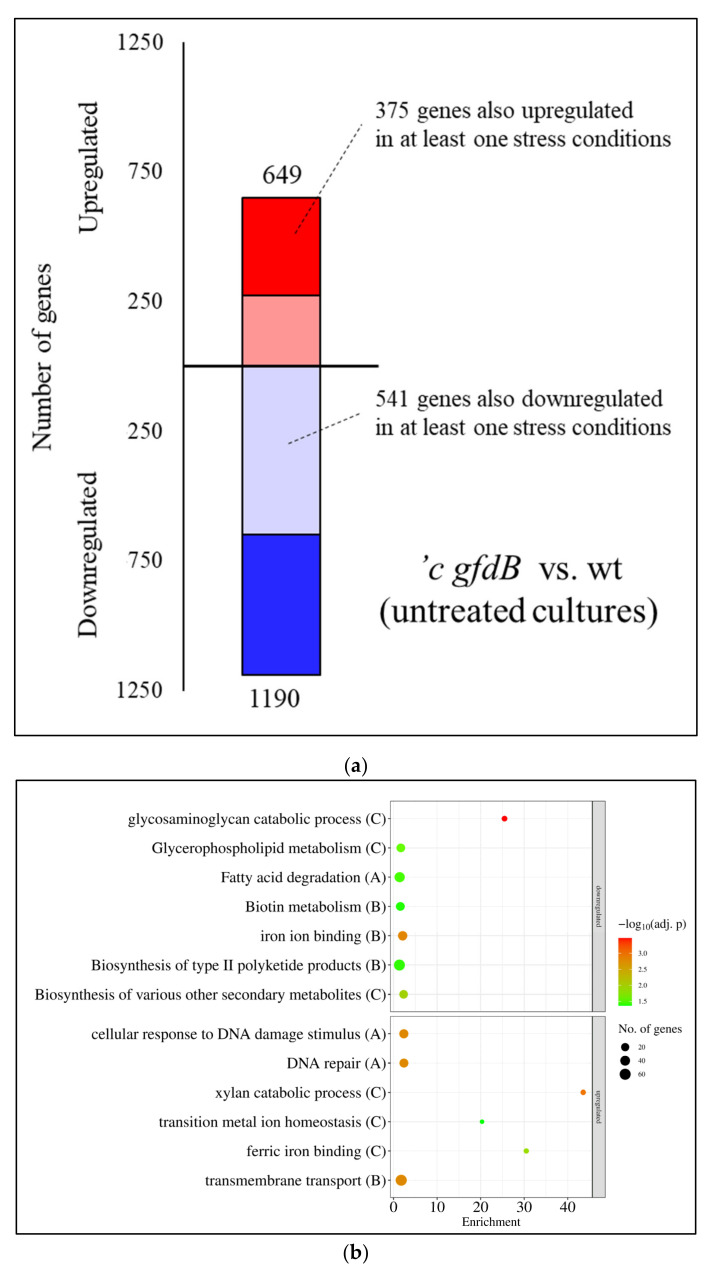
Direct comparison of the transcriptomes of *A. wentii* CBS141173 and ′c *gfdB* strains under untreated conditions. The number of upregulated (red) and downregulated (blue) genes (DEGs with |log_2_FC| > 1) are presented from the comparison of the ′c *gfdB* mutant vs. the CBS141173 strain in untreated cultures (**a**). Gene set enrichment analyses of the consequences of *A. nidulans-gfdB* expression in *A. wentii* under untreated conditions (**b**). Selected significantly enriched (*p* adjusted < 0.05) GO and KEGG pathway terms are presented. The full list of the enriched terms are available in Table S3. Letters in parentheses indicate the studied gene set: “A”—all DEGs; “B”—DEGs with |log_2_FC| > 1; “C”—DEGs with |log_2_FC| > 2. If a selected term was enriched in more than one gene set, only the set with the strongest criteria is presented.

Direct comparison of the transcriptomes of the *An-gfdB* expressing mutant and the reference strain of *A. wentii*, under untreated conditions, revealed much bigger differences (Figure 8a) than those found in the case of *A. nidulans* (Figure 5a). The overlap between the upregulated or downregulated gene sets of the mutant and the reference strain found in each treatment, as well as the correlation between the transcriptional changes of the two strains caused by the same treatment, were also smaller in *A. wentii* (Figures S4 and S5) than in *A. nidulans* (Figures S2 and S3). The expression of the *gfdB* gene in *A. wentii* caused bigger alteration in the genome-wide transcription profiles than did the deletion of this gene in *A. nidulans*. This is in line with the observation that the expression of *gfdB* significantly reduced the growth of *A. wentii* (Figure 1b), while the deletion of this gene had no significant effect on the growth in *A. nidulans* (Figure 1a).

Expression of the *An-gfdB* gene in *A. wentii* resulted in the upregulation of 649 genes and the downregulation of 1190 genes under untreated conditions relative to the reference strain (Figure 8a, Table S5). Genes that showed upregulation in at least one stress treatment in the CBS141173 reference strain were significantly enriched in the upregulated gene set (375 genes out of the 649 genes), while genes that showed downregulation in at least one stress treatment in the reference strain were significantly enriched in the downregulated gene set (541 genes out of the 1190 genes) (Figure 8a, Table S5). The genome-wide transcriptional changes induced by *gfdB* gene expression showed some positive correlation with the treatment-elicited transcriptional changes of the reference strain (Figure 6c). 

Regarding the results of the gene set enrichment analyses, the secondary metabolism genes and biotin metabolism genes were enriched in the downregulated gene set, while the DNA repair and transporter genes were enriched in the upregulated gene set under untreated conditions when the transcriptomes of the ′c *gfdB* mutant and the CBS141173 reference strain were compared (Figure 8b).

## 4. Discussion

### 4.1. A. nidulans THS30 Showed Canonic Hyperosmotic Stress Response under Sorbitol or NaCl Stress

*A. nidulans* THS30 is an osmotolerant, but not an osmophilic, strain [22]. Consequently, both sorbitol and NaCl reduced its growth (Figure 1a), which was accompanied by the downregulation of the mitotic cell cycle, DNA replication, and the translation genes (Figure 4, Table S3) and the upregulation of several genes characteristic for hyperosmotic stress, including elements of the high-osmolarity glycerol (HOG) map kinase pathway (*pbsA*, *hogA*, *ptpA*, *atfA*) [39,43], as well as glycerol (*gfdB*) [48] and trehalose (*tpsA*, *orlA*, *treB*) [49,50] metabolism genes in both treatments (Figure 4, Tables S3 and S4). Moreover, the upregulation of transporter and protein folding genes, transcriptional alterations in the developmental process (conidiogenesis, sexual reproduction) genes (Figure 4, Table S3) are not unexpected under hyperosmotic stress [13,29,30,51,52,53]. The activation of DNA repair, protein degradation processes, and autophagy are common elements of responses to significant stresses [54,55]. Their downregulation under osmotic stress (observed after sorbitol, in part after NaCl, and also after the combined NaCl + sorbitol stress treatments (Figure 4, Table S3) is unexpected, and elucidating their importance requires further investigations. Nevertheless, these changes suggest that the applied treatments were not significant for *A. nidulans* THS30, which is in line with the good osmotolerance of the strain [22].

### 4.2. The Combination of Sorbitol and NaCl Stress Treatments Was Antagonistic

Surprisingly, the stress response to the combined NaCl + sorbitol treatment was much weaker than when NaCl or sorbitol was applied alone to the *A. nidulans* THS30 cultures (Figure 1a and Figure 2a). 

No growth reduction (Figure 1a) and no upregulation of “osmotic stress specific genes” (but with *sskA* encoding a response regulator of the HOG pathway [40]) were observed (Figure 4, Tables S3 and S4). The three other strains studied also showed a weak stress response under the combined NaCl + sorbitol treatment relative to that of the single stressor treatments (Figure 1, Figure 3 and Figure 6). Both sorbitol and Na^+^, as osmotically active compounds [19], cause hyperosmotic stress at high concentrations. Cells usually protect themselves against the high osmolarity of the environment by synthetizing osmolytes and/or taking up such compounds from the environment [21,29,30]. Sorbitol and NaCl are kosmotropes (compounds that stabilize the structure of macromolecules) [19], and Na^+^ is also toxic at high concentrations [30,56]. In *S. cerevisiae*, Na^+^ inhibits the Hal2 nucleotidase involved in sulfate assimilation [57] and over-activate the Gcn2-Gcn4 system regulating amino acid metabolism [30]. As an explanation for the antagonistic interaction between sorbitol and NaCl, it is hypothesized that Na^+^ ions reduced the osmotic gradient by entering the cells in a rapid and uncontrolled manner [58], while sorbitol, which also entered the cells (taken up by the cells), but more slowly than Na^+^, somehow protected proteins from Na^+^ toxicity. (During sorbitol treatment, because of the slow sorbitol uptake, the osmotic gradient did not decrease fast enough for efficient protection, while under NaCl stress, although the quick Na^+^ influx decreased the osmotic gradient substantially, cells were exposed to high Na^+^ toxicity.) In line with our hypothesis, de Vries et al. [21] showed that osmotic stress-sensitive Δ*gldB A. nidulans* mutants were able to compensate for NaCl stress by taking up extracellularly added polyols (glycerol, erythritol, arabitol, mannitol), while de Lima Alves et al. [19] experimentally demonstrated that kosmotropic compounds (including sorbitol) can neutralize the effect of chaotropic agents in in vitro agar-gelation assays.

### 4.3. The Responses of A. wentii CBS141173 to Sorbitol and NaCl Were Not Canonic Hyperosmotic Stress Responses

*A. wentii* CBS141173 is an osmophilic strain [19,22,28]. As mentioned above, it grew better in the presence of 1 M sorbitol or 2 M NaCl on agar plates than without these additives [28]. In our experiments, sorbitol treatment increased growth as it was expected (Figure 4a), however NaCl treatment decreased it (Figure 4a). This growth reduction may be explained by the toxicity of Na^+^ [30,56], which may have been more pronounced in submerged than in surface cultures. In line with the osmophilic nature of *A. wentii*, the observed responses were not canonic hyperosmotic stress responses. Bulk upregulation of orthologues of *A. nidulans* HOG pathway genes (e.g., upregulation of *pbsA*, *ptpA*, *hogA* orthologues), trehalose biosynthesis genes, or glycerol metabolism genes (Table S4) were not observed. Mitochondrial functions were downregulated by sorbitol treatment in *A. wentii* (Figure 7, Table S3), while it was upregulated in *A. nidulans* (Figure 4, Table S3). Bulk downregulation of mitotic cell cycle, and DNA replication genes were observed only in *A. nidulans* (Figure 4, Table S3), but not in *A. wentii* (Figure 7, Table S3).

### 4.4. GfdB–Easier to Lose Than to Get Back

In *A. nidulans*, glycerol is synthetized by either the glycerol-3-phosphate (GfdA/GfdB) pathway (dihydroxyacetone-phosphate → glycerol-3-phosphate → glycerol) or the dihydroxyacetone (NADP-specific glycerol dehydrogenase) pathway (dihydroxyacetone-phosphate → dihydroxyacetone → glycerol). Although both pathways were active during the NaCl-elicited early stress response [48], the phenotype of the gene deletion mutants showed the dominance of the dihydroxyacetone pathway [21]. Importantly, in stress adapted cultures, only the glycerol-3-phosphate pathway was found to be crucial [48]. Fillinger et al. [25] also revealed that GfdA is responsible for almost the entire extent of NAD-specific glycerol-3-phosphate dehydrogenase activity at normal osmolarity [25]. The strong upregulation of *gfdB* under both sorbitol and NaCl stress treatments (Figure 2, Table S4) demonstrates that this gene (and as a consequence, the glycerol-3-phosphate pathway) was important in the adaptation to increased osmolarity. Interestingly, the deletion of *gfdB* resulted in minor changes in the stress responses (Figure 3), growth (Figure 1), and osmotic stress tolerance [24] of *A. nidulans*. This phenotype may be explained by the complexity of glycerol metabolism: in the absence of GfdB, both GfdA and the NADP-specific glycerol dehydrogenase pathway can provide enough glycerol for osmoadaptation. Moreover, the deletion of *gfdB* in *A. nidulans* THS30 resulted in transcriptional changes, with some similarity to those elicited by hyperosmotic stress (Figure 3c). These changes may prepare the mutant to adapt more easily to hyperosmotic stress, which may also explain the unchanged osmotolerance of the Δ*gfdB* strain. These properties of the *A. nidulans* Δ*gfdB* strain suggest that mutants with GfdB deficiency are not necessarily under strong selection pressure caused by the impaired osmoregulation. Nevertheless, the *A. nidulans* Δ*gfdB* strains possessed elevated oxidative stress sensitivity [24], demonstrating that losing *gfdB* does have negative consequences, which may stabilize the presence of this gene in the genome. 

The expression of *An-gfdB* in *A. wentii* did not eliminate the osmophily of this species, according to the growth pattern of the mutant: the presence of *An-gfdB* decreased growth both under high and normal osmolarity and did not prevent the growth-eliciting effect of sorbitol (Figure 1b). Previous studies using agar plate cultures also showed that although expression of *An-gfdB* decreased the growth-promoting properties of the sorbitol and NaCl treatments, the mutant strains did not grow better than the reference strain under low osmolarity [28]. Nevertheless, the presence of GfdB disturbed the physiology of this species. 

The transcriptome of the *A. wentii* ′c *gfdB* mutant differed more from the CBS141173 reference strain (Figure 8a and Figure S1) than did that of the *A. nidulans* Δ*gfdB* mutant from the THS30 strain (Figure 5a and Figure S1). The genome-wide transcriptional responses of the mutant and the wild type strains elicited by the high osmolarity treatments differed more and correlated less in the case of *A. wentii* (Figure 6c and Figure S4) than in *A. nidulans* (Figure 3c and Figure S2). These changes may be the consequences of the improper regulation of *gfdB* (Figure 2c) caused by either the inappropriate recognition of the *A. nidulans* promoter in *A. wentii* and/or the epigenetic environment of the gene insertion site. The changes in *gfdB* transcription may have resulted in glycerol-3-phosphate dehydrogenase activities in excess of what is required. Our data for the ′c *gfdB* strain suggest that after evolutionary adaptation to the loss of GfdB, any processes that increase Gfd activity in excess (e.g., duplication of *gfdA* or horizontal gene transfer of a *gfdB* gene) can be disadvantageous. However, the question regarding whether the loss of *gfdB* was followed by the development of the osmophilic property of the species or vice versa remains unanswered. Glycerol is needed for various biosynthetic processes (e.g., phospholipid biosynthesis), to regulate intracellular osmotic concentration and even to maintain the appropriate redox milieu [29]. Under conditions when the osmolarity of the environment can change frequently, a fine-tuning of glycerol synthesis can be essential, which may create a need for complex glycerol metabolism, including two different glycerol biosynthesis pathways [25,48] and two differently regulated glycerol-3-phosphate dehydrogenase genes [24]. Under continuously high osmolarity (an unchanging environment), the cost of this complexity may offset its reduced benefits. Therefore, we assume that the lack of the *gfdB* gene in some *Aspergillus* species, like *A. wentii*, is most likely not the cause of osmophily, but rather the consequence of the adaptation to an environment where the osmolarity is constantly high. 

Although the biotechnological application of xerophilic/osmophilic species within the family *Aspergillaceae* and their valuable enzymes and secondary metabolites is dynamically expanding [59,60,61,62], the mechanism of their adaptation to high osmolarity has been studied in detail in only a few species, including *Aspergillus ruber* [1], *Xeromyces bisporus* [63], and *Aspergillus sydowii* [64]. *A. wentii* is a good lipase [65], a valuable competitive solute (glycerol, erythritol, mannitol; [19]), and an antioxidant [66] producer fungus, with possible future applications in biodiesel production [67]. Furthermore, *A. wentii* is also a mycotoxigenic (e.g., emodin; [68]) and a potential opportunistic human pathogenic [69] mold. Hopefully, this comparative transcriptomics study will help experts working on either the further development of *A. wentii* as a promising future workhorse in the fermentation industry, or on the control of its disadvantageous mycotoxin production and pathogenicity under both normal and high osmolarity conditions.

Manipulating the stress tolerance of fungi is an effective way to increase their applicability in industry. Deletion or overexpression of some of their own genes, or heterologous expression of foreign genes, are routinely used tools to improve their stress tolerance attributes. Our results show that these approaches can sometimes be challenging. Because of the flexibility of their cellular regulatory network, fungi can often easily compensate for the loss of a single stress-response gene. On the other hand, the heterologous expression of a new gene, with its own promoter, may be more detrimental than expected when the fungi hosting the gene are unable to regulate it properly. Therefore, a deeper understanding of the stress adaptation processes and the physiological consequences of these genetic manipulations is crucially important in designing and constructing higher-performing mutants. In summary, the identification of genes that potentially enhance stress tolerance and the introduction of these genes into the genome of the host organism, as well as the selection of promoters with appropriate binding sites for the regulatory elements required for the optimal functioning of the foreign gene, are equally important for successful strain development. Future *A. wentii* strain improvements should therefore include the selection of appropriate *A. wentii* promoter and terminator sequences for the constitutive expression of the *A. nidulans gfdB* gene [28].

## Figures and Tables

**Table 1:** Strains used in the study.

Strain	Genotype	Reference
*A. nidulans* THS30	*pyrG89*, *AfupyrG^+^*; *pyroA^+^*; *veA^+^*	[32]
*A. nidulans* Δ*gfdB*	*pyrG89*; Δ*gfdB*::*AfupyrG*^+^; *pyroA*^+^; *veA*^+^	[24]
*A. wentii* CBS141173	Wild-type	[22]
*A. wentii* ′c *gfdB*	CBS141173 strain harboring one copy of *A. nidulans gfdB* with its own promoter and terminator sequences and *HygR* as a selection marker gene	[28]

## Data Availability

The transcriptome datasets are available in the Gene Expression Omnibus database (GEO; http://www.ncbi.nlm.nih.gov/geo/) under the following accession number: GSE255841.

## Supplementary Materials

The following supporting information can be downloaded at: https://www.mdpi.com/article/10.3390/jof10040291/s1. Figure S1: Principal component (PC) analysis of the RNAseq data obtained from stress treated and untreated cultures of *A. nidulans* (A) and *A. wentii* (B) strains; Figure S2: Overlap between the stress responses of the *A. nidulans* THS30 and the Δ*gfdB* strains; Figure S3: Gene set enrichment analyses of the effect of sorbitol (A), NaCl (B), and NaCl + sorbitol (C) treatments on *A. nidulans* Δ*gfdB*; Figure S4: Overlap between the stress responses of the *A. wentii* CBS141173 and the ′c *gfdB* strains; Figure S5: Gene set enrichment analyses of the effect of sorbitol (A), NaCl (B), and NaCl + sorbitol (C) treatments on *A. wentii* ′c *gfdB*; Table S1: Primer pairs used in the study; Table S2: RT-qPCR data of the *A. wentii* and *A. nidulans* cultures; Table S3: Results of the gene set enrichment analyses; Table S4: RNAseq data of “hyperosmotic stress”, “oxidative stress”, and “glycerol metabolism” genes in *A. nidulans*, as well as the RNAseq data for their *A. wentii* orthologues; Table S5: Transcriptional behavior of selected genes under the applied osmotic stress conditions.
