# Supplementary material for: Aspergillus nidulans gfdB, Encoding the Hyperosmotic Stress Protein Glycerol-3-phosphate Dehydrogenase, Disrupts Osmoadaptation in Aspergillus wentii
Source: J Fungi (Basel). 2024 Apr 16;10(4):291. doi: 10.3390/jof10040291 (PMC11051529; doi:10.3390/jof10040291)
Supplement: Supplementary file 1 [file jof-10-00291-s001.zip › Supplementary Figures.pdf]

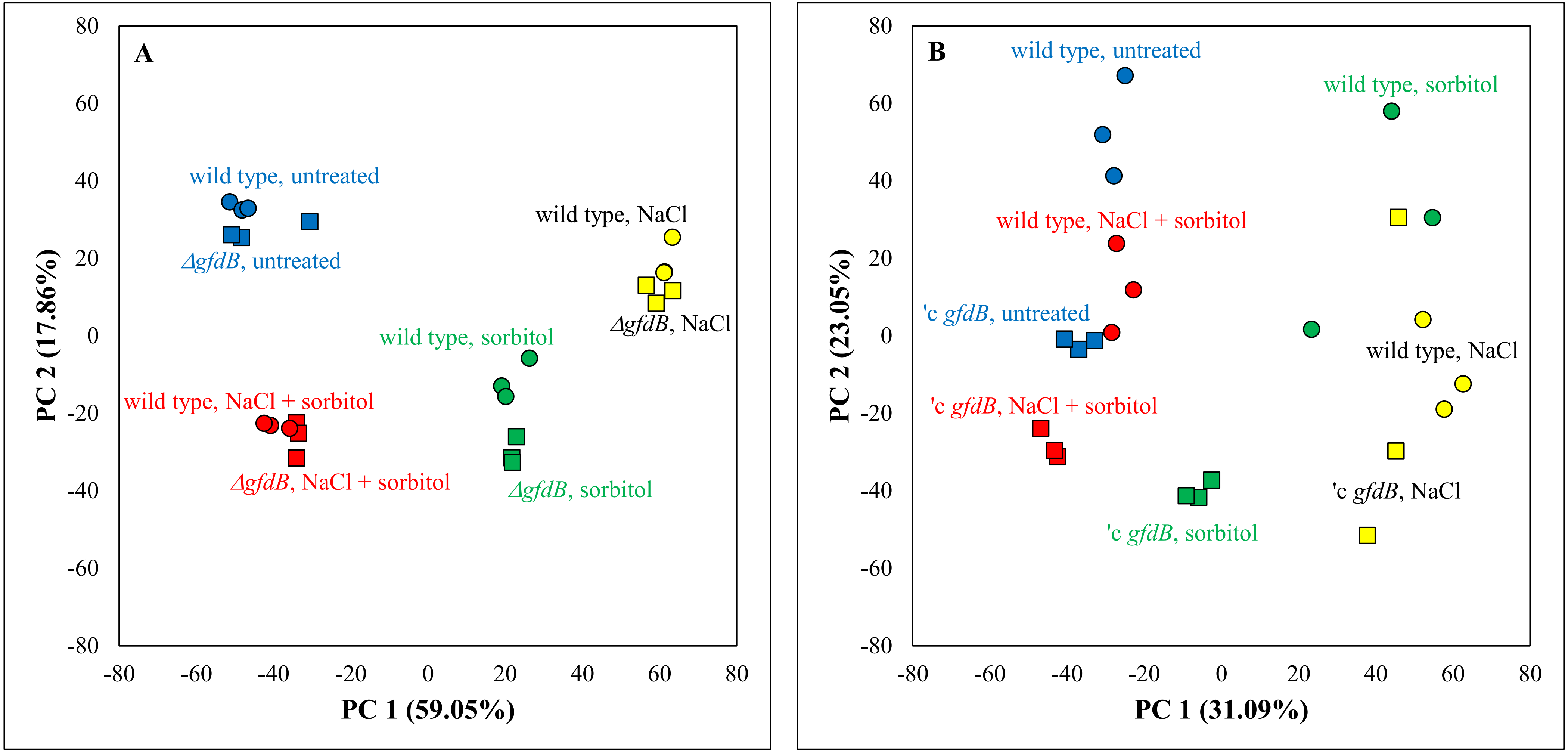

Figure S1 Principal component (PC) analysis of the RNAseq data obtained from stress treated and untreated cultures of *A. nidulans* (A) and *A. wentii* (B) strains. The analysis was carried out using the rlog data generated by the DESeq2 software.

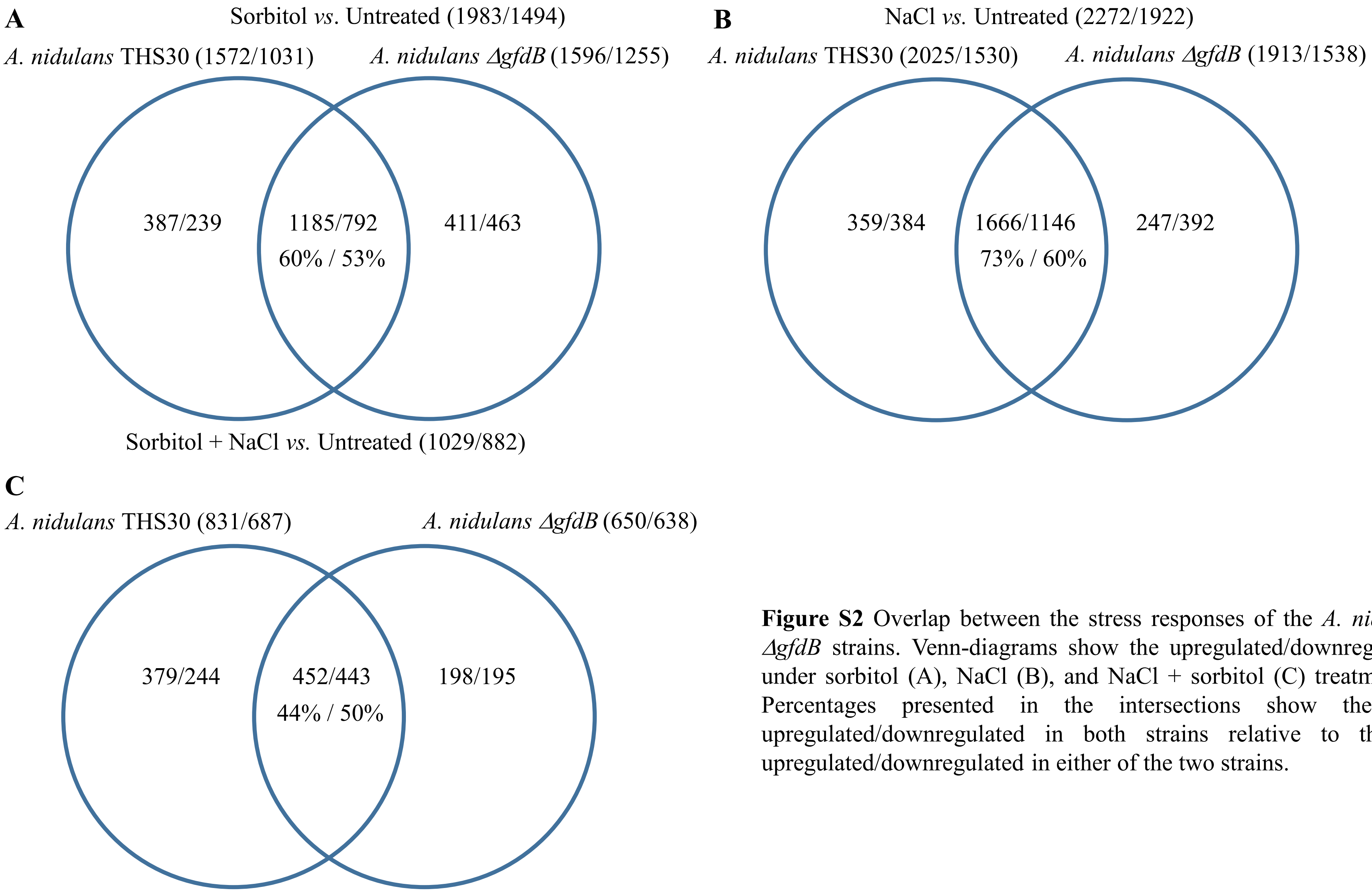

Figure S2 Overlap between the stress responses of the *A. nidulans* THS30 and the *ΔgfdB* strains. Venn-diagrams show the upregulated/downregulated genes observed under sorbitol (A), NaCl (B), and NaCl + sorbitol (C) treatments in the two strain. Percentages presented in the intersections show the number of genes upregulated/downregulated in both strains relative to the number of genes upregulated/downregulated in either of the two strains.

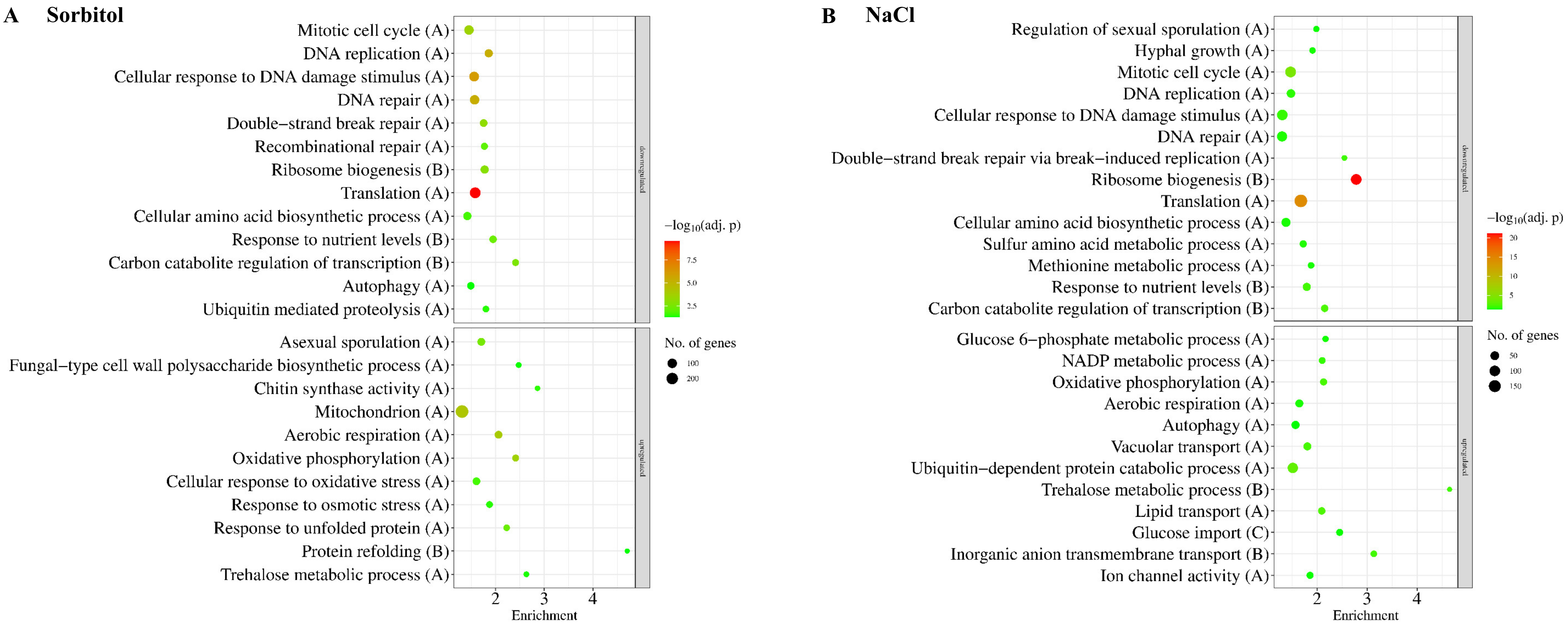

Figures S3A and S3B Gene set enrichment analyses of the effect of sorbitol (A) and NaCl (B) treatments on *A. nidulans ΔgfdB*.
Selected significantly enriched (*p* adjusted < 0.05) GO and KEGG pathway terms are presented. The full list of the enriched terms are available in Table S3. Letters in parentheses indicate the studied gene set: "A"— all DEGs, "B"— DEGs with $|log_2FC| > 1$, "C"— DEGs with $|log_2FC| > 2$. If a selected term was enriched in more than one gene set, only the set with the strongest criteria is presented.

C NaCl + sorbitol

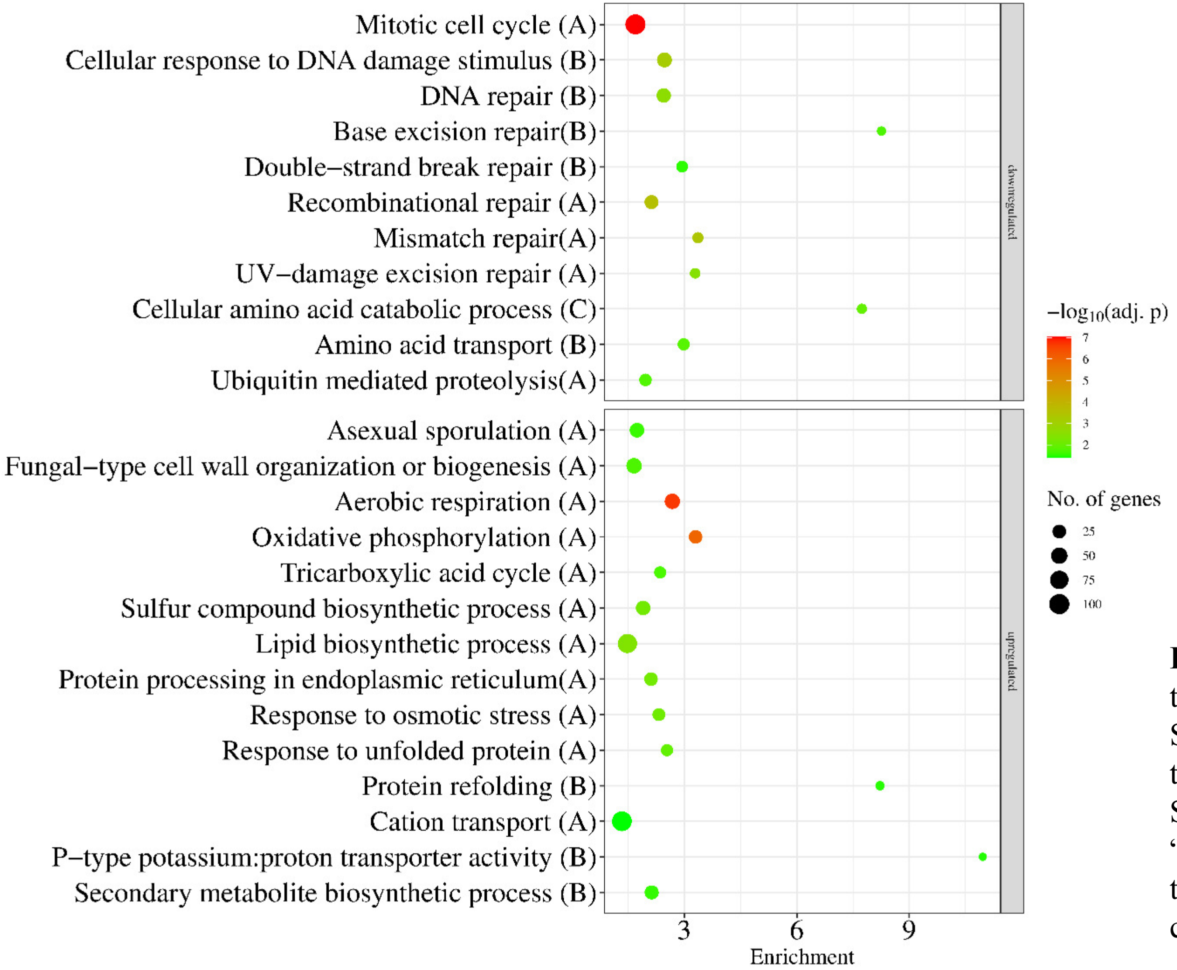

Figure S3C Gene set enrichment analyses of the effect of NaCl + sorbitol (C) treatment on *A. nidulans ΔgfdB*.
Selected significantly enriched (p adjusted < 0.05) GO and KEGG pathway terms are presented. The full list of the enriched terms are available in Table S3. Letters in parentheses indicate the studied gene set: "A"— all DEGs, "B"— DEGs with $|log_2FC| > 1$, "C"— DEGs with $|log_2FC| > 2$. If a selected term was enriched in more than one gene set, only the set with the strongest criteria is presented.

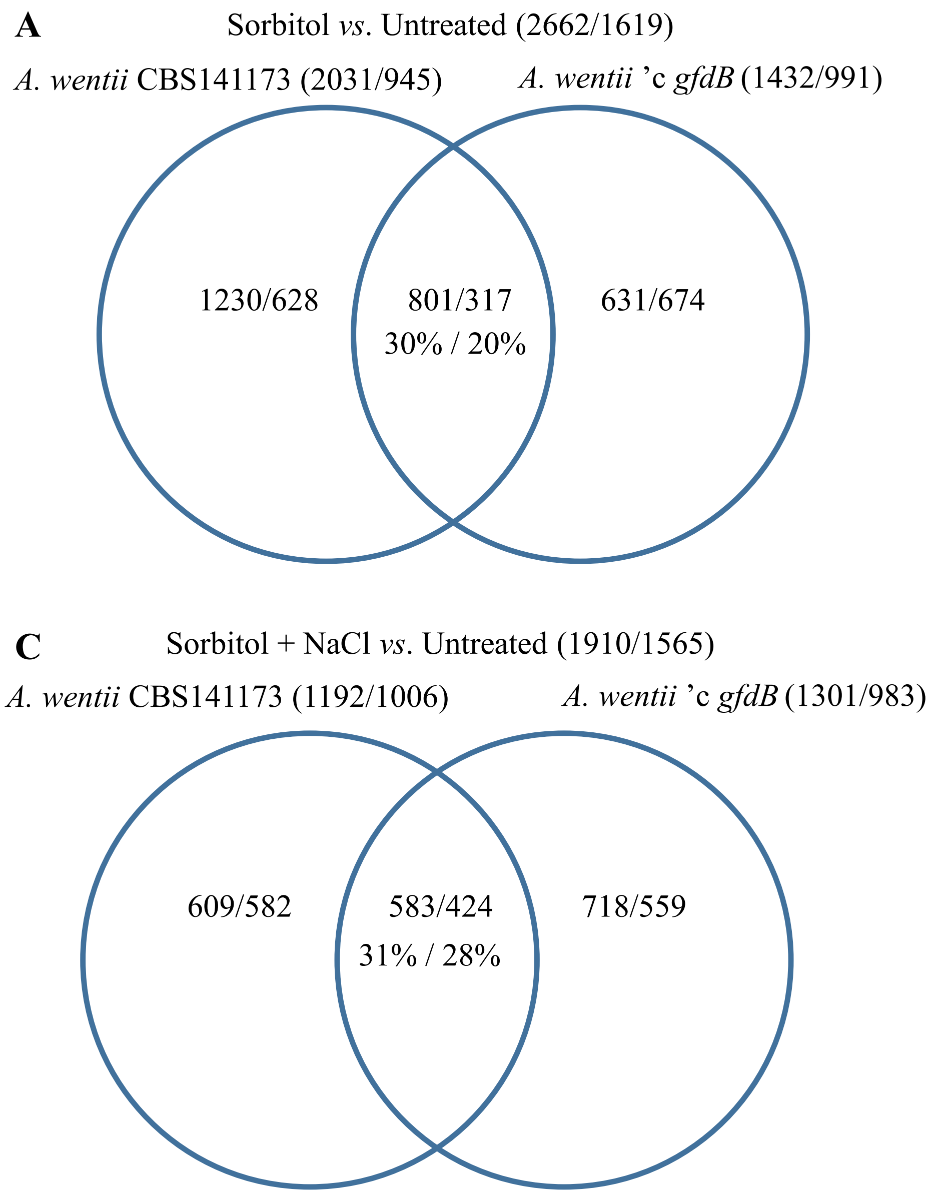

Figure S4 Overlap between the stress responses of the *A. wentii* CBS141173 and the ’c *gfdB* strains. Venn-diagrams show the upregulated/downregulated genes observed under sorbitol (A), NaCl (B), and NaCl + sorbitol (C) treatments in the two strain. Percentages presented in the intersections show the number of genes upregulated/downregulated in both strains relative to the number of genes upregulated/downregulated in either of the two strains.

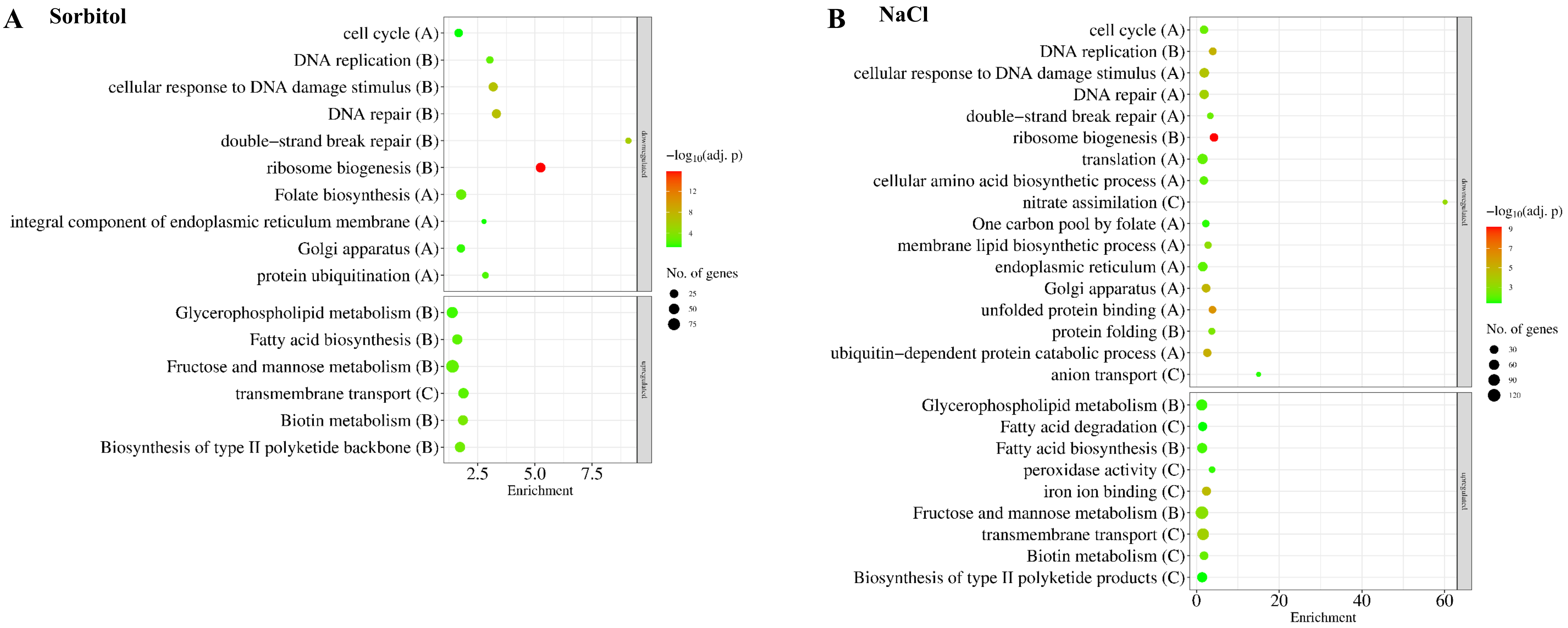

Figures S5A and S5B Gene set enrichment analyses of the effect of sorbitol (A) and NaCl (B) treatments on *A. wentii* ’c *gfdB*.
Selected significantly enriched (p adjusted < 0.05) GO and KEGG pathway terms are presented. The full list of the enriched terms are available in Table S3. Letters in parentheses indicate the studied gene set: “A”— all DEGs, “B”— DEGs with $|log_2FC| > 1$, “C”— DEGs with $|log_2FC| > 2$. If a selected term was enriched in more than one gene set, only the set with the strongest criteria is presented.

C NaCl + sorbitol

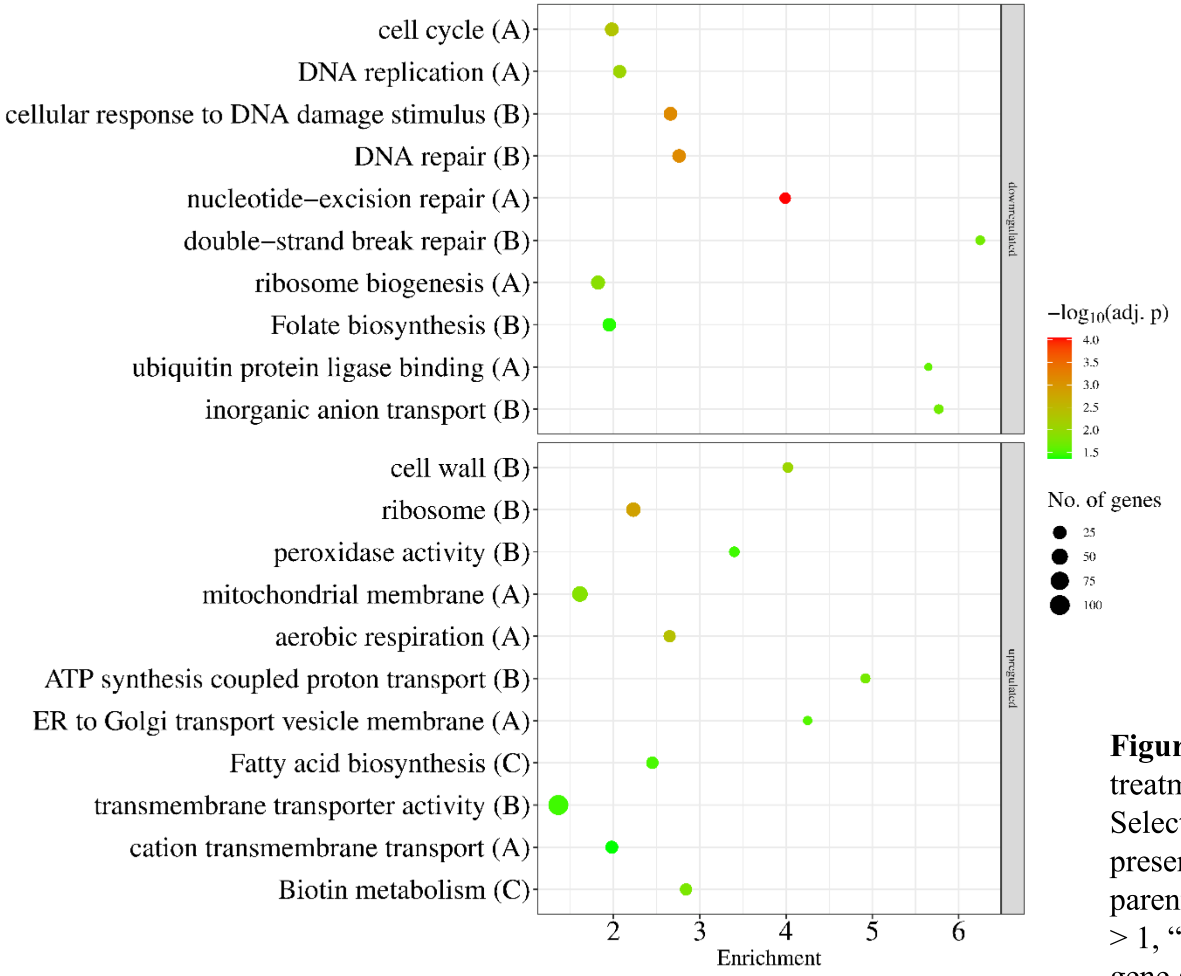

Figure S5C Gene set enrichment analyses of the effect of NaCl + sorbitol (C) treatment on *A. wentii* ’c *gfdB*.
Selected significantly enriched (*p* adjusted < 0.05) GO and KEGG pathway terms are presented. The full list of the enriched terms are available in Table S3. Letters in parentheses indicate the studied gene set: “A”— all DEGs, “B”— DEGs with $|log_2FC| > 1$, “C”— DEGs with $|log_2FC| > 2$. If a selected term was enriched in more than one gene set, only the set with the strongest criteria is presented.
