# Supplementary material for: Aspergillus nidulans gfdB, Encoding the Hyperosmotic Stress Protein Glycerol-3-phosphate Dehydrogenase, Disrupts Osmoadaptation in Aspergillus wentii
Source: J Fungi (Basel). 2024 Apr 16;10(4):291. doi: 10.3390/jof10040291 (PMC11051529; doi:10.3390/jof10040291)
Supplement: Supplementary file 1 [file jof-10-00291-s001.zip › Table S1.pdf]

Table S1 Primer pairs used in the study

Aspergillus nidulans

AN0351
5’-ATCCTGCCTCTTCTCTTTG-3’
5’-ACTCCTTCCTCATCCACATC-3’

AN0443
5´-GGGCGATGAGTTGTTCTG-3´
5´-TTCAGGCGGAGTAGTTGG-3´

AN1679
5´-TGTCTCTTCCTTCCCATTTC-3´
5´-ACCATTGCCAACTTCAGTC-3´

AN2815
5´-AAACTGCTCCCGCTGACATC-3´
5´-ACGACTTTGACGCCAACCTTC-3´

AN4829
5´-CTCAACCACAACCATTCAG-3´
5´-CCCTTTTCCTTAGACCATTC-3´

AN5523
5’-CTGCCAACCACTCCTAATG-3’
5’-GTCAACGCCTACCATAAGC-3’

AN5634
5’-GGTGGTCTTACTGCTGTGATG-3’
5’-CAATGGTGGATGTGATGAGGG-3’

AN5885
5´-CCACCCCAGCAGATTATTAC-3´
5´-TCCCCTTGATTCCCATTCC-3´

AN5975
5´-ATGAGGCTGGCTACGAAG-3´
5´-AGTGAGTGGGGTGGAAAC-3´

AN6542
5′-GAAGTCCTACGAACTGCCTGATG-3’
5′-AAGAACGCTGGGCTGGAA-3’

AN6700
5’-CCTATTCCCGAGCAAGTTC-3’
5’-TGATGTTCCTGACGATGGC-3’

AN6792
5´-ACCGTCCCTTCCGATTCTCC-3´
5´-AGCGTCACCTTCCCGTCTG-3´

AN8639
5´-CACTCCGTCCCCTTCACTG-3´
5´-GTCGCTGCCTTGTAAACCG-3´

AN8803
5’-CAACAAGGGCAACAGCAAC-3’
5’-GAGGACAGCAACATCAAGC-3’

Aspergillus wentii

ASPWEDRAFT_105168
5´-CATCACCCCCCAGACATC-3´
5´-GCGACCGTCAGAGAACTTG-3´

ASPWEDRAFT_117545
5´-ACCAAGACCATCACCAACTAC-3´
5´-GCATTCTCACAAGCAATCAC-3´

ASPWEDRAFT_117870
5´-ATGTCGCCTCGCATTCTG-3´
5´-GGGTTGTCTCGCCATTGAG-3´

ASPWEDRAFT_121910
5´-GCCAGGTTCAGTTTTCTTCG-3´
5´-CATATCGCCCGTCTCAATG-3´

ASPWEDRAFT_165285
5´-TTTCCTCTTCGCCTCTTTC-3´
5´-GTCGTCAATCATCTCAATGG-3´

ASPWEDRAFT_166217
5´-CGGTGGTCTTATTGATGC-3
5´-TTGTCGGGGAGTGTGTAG-3´

ASPWEDRAFT_DRAFT_167845
5´-TACTGCCATTGCCATTGTC-3´
5´-CCTTCTTGACCTCGTTCTTG-3´

ASPWEDRAFT_168397
5´-TCTGGTGACGGTGTTACTC-3´
5´-ATCTCCTGCTCAAAGTCG-3´

ASPWEDRAFT_173702
5´-GATGGAGACGGTATGCTG-3´
5´-GCCTTCTTGGTATTCTTGC-3´

ASPWEDRAFT_184430
5´-GGGAATGAAAGGGAAGTTG-3´
5´-TTGTGGAGTGGCTGAATG-3´

ASPWEDRAFT_189665
5´-CCAAGGGAGAGACTGATG-3´
5´-GTAGGAGGAAAGCGAAAAC-3´

ASPWEDRAFT_25409
5´-GTCCCTCTGCCCACTTTG-3´
5´-AACGACACGCTCACCAATC-3´

ASPWEDRAFT_33357
5´-TCTTCACGCAGGTTATCTCG-3´
5´-AGGTCTTTGGGGATGTTGG-3´

ASPWEDRAFT_35534
5´-CAGGGTGTGCGTGTATTGG-3´
5´-GTGTTGGTGGGAGAAGTTGG-3´
